# Supplementary figures and images for: The characteristics of early-stage research into human genes are substantially different from subsequent research
Source: PLoS Biol. 2022 Jan 6;20(1):e3001520. doi: 10.1371/journal.pbio.3001520 (PMC8769369; doi:10.1371/journal.pbio.3001520)

Figure S1

A

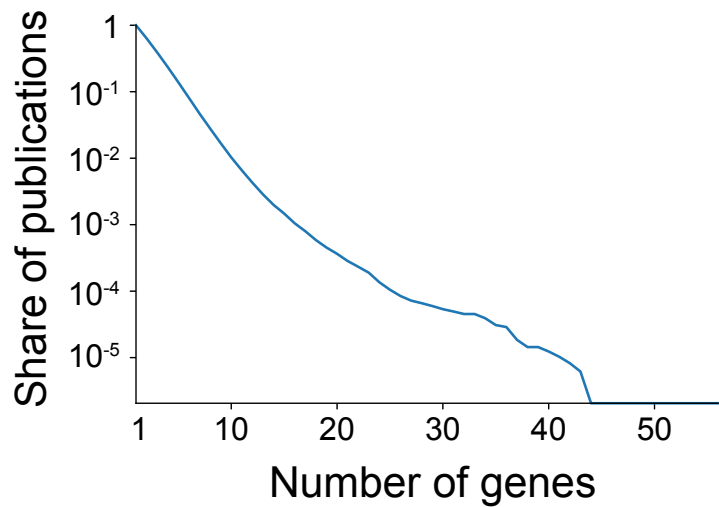

B

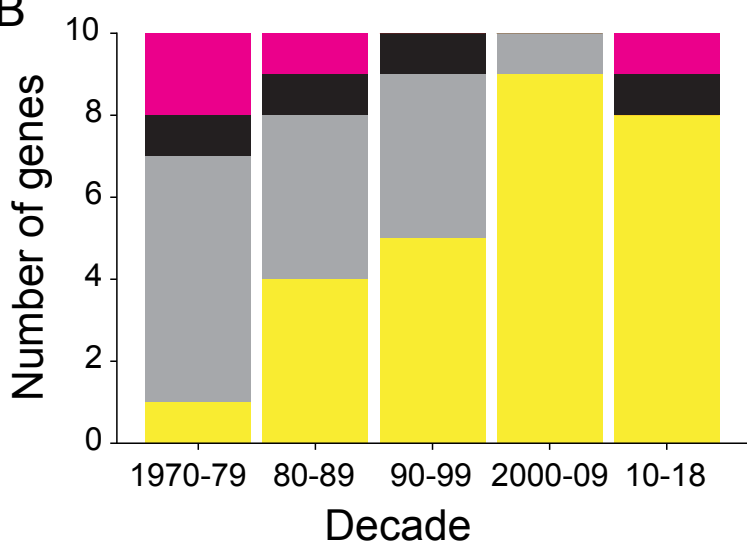

Supplement: S1 Fig — (A) Survival analysis share of publications highlighting a gene in the title or abstract that highlight at least the indicated number of genes. (B) Estimation of impact of changes in gene nomenclature and other issues in determining whether a given gene is highlighted in a publication or not. For each decade, we randomly selected 10 genes that our automated approach did not identify as being highlighted by name in the title or abstract prior to the year of the oldest automatically identified publication. We mark in yellow “clear cases,” where past nomenclature aligns with today’s nomenclature and for which we could not find any preceding highlighting. We mark in gray “foggy cases,” where we found that entities related to those genes (e.g., symptoms, enzymatic activities,…) had been highlighted before, but—in contrast to some other genes—are not currently accepted as namesakes or synonyms of these genes by NCBI Gene. We mark in pink “missed by name cases,” where the gene highlighted in a publication was misattributed because the 2 genes either swapped names later or the name of one gene became part of the name of the other gene. Finally, we mark in black “missed cases,” where an earlier highlighting of the genes was missed by our automated computational approach. Combines data from MEDLINE, NCBI gene and taxonomy information, gene2pubmed, and PubTator. For data underlying the figure, see https://doi.org/10.21985/n2-b5bm-3b17. (PDF) [file pbio.3001520.s002.pdf]

Figure S2

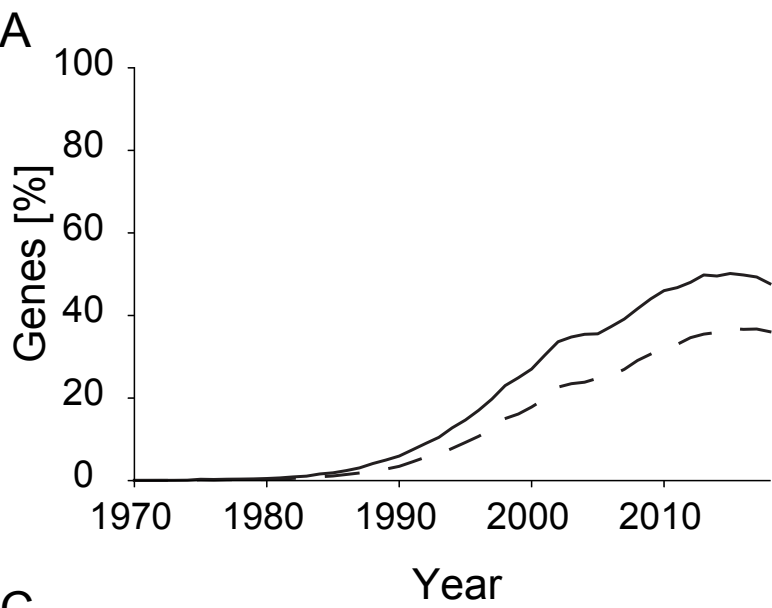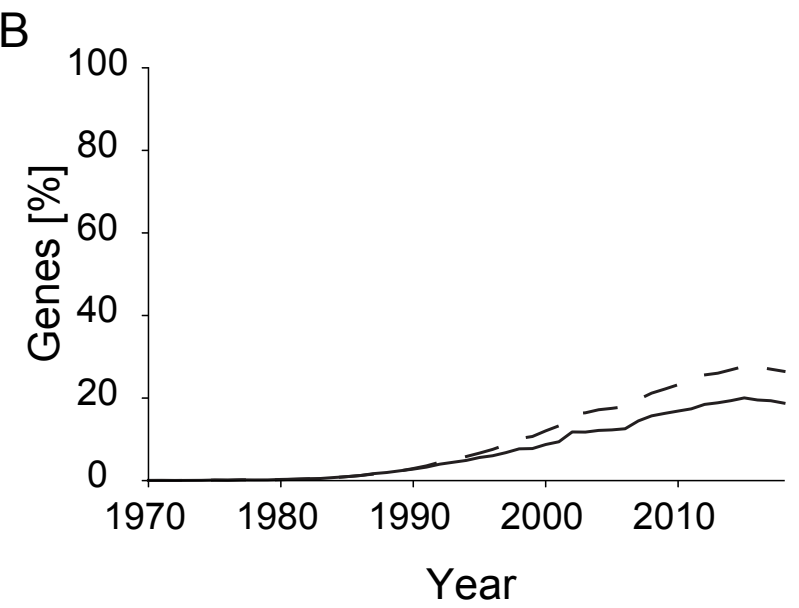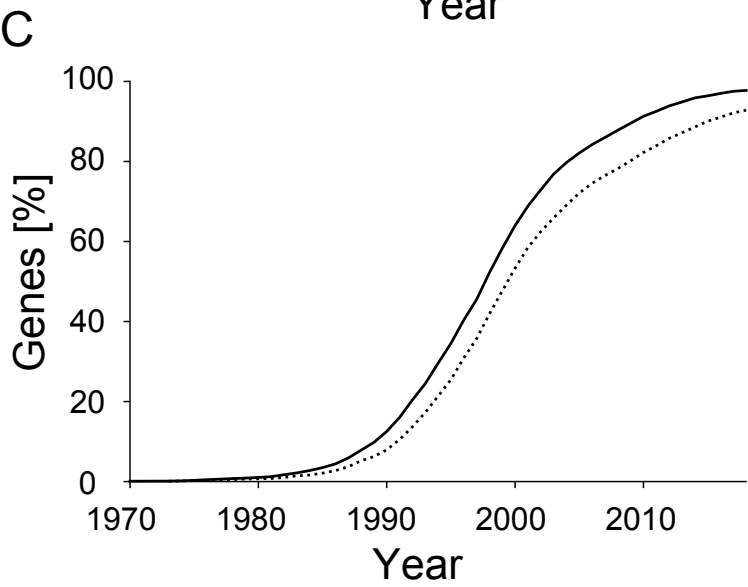

Supplement: S2 Fig — (A) Percentage of protein-coding genes highlighted in a given year in at least 1 publication. (B) Percentage of protein-coding genes highlighted in a given year in at least 1 publication that highlights a single gene. (C) Share of human loss-of-function intolerant protein-coding genes which have been highlighted (mentioned by name in title or abstract) until the indicated year (solid) or mentioned by name in the title (dotted). Combines data from MEDLINE, NCBI gene and taxonomy information, ene2pubmed, PubTator, and Karczewski and colleagues. [32]. For data underlying the figure, see https://doi.org/10.21985/n2-b5bm-3b17. (PDF) [file pbio.3001520.s003.pdf]

Figure S3

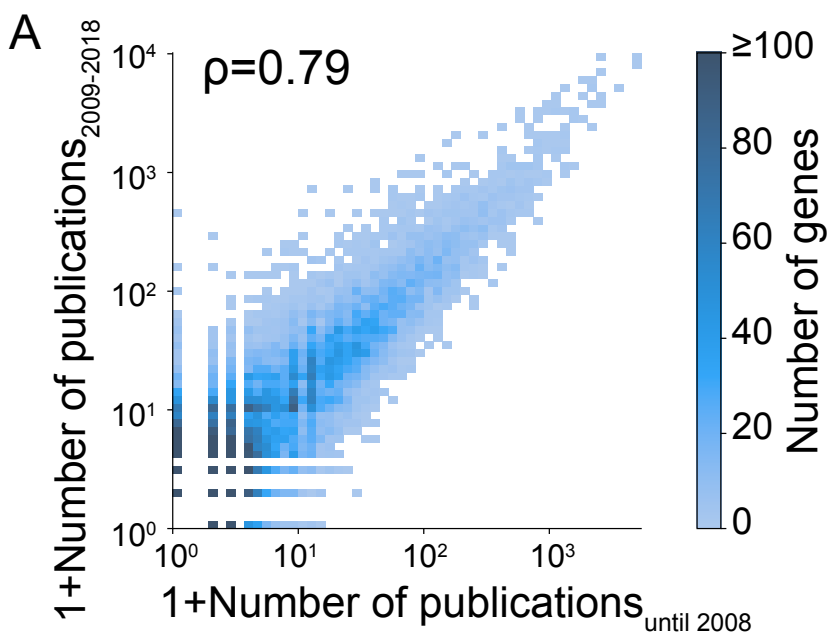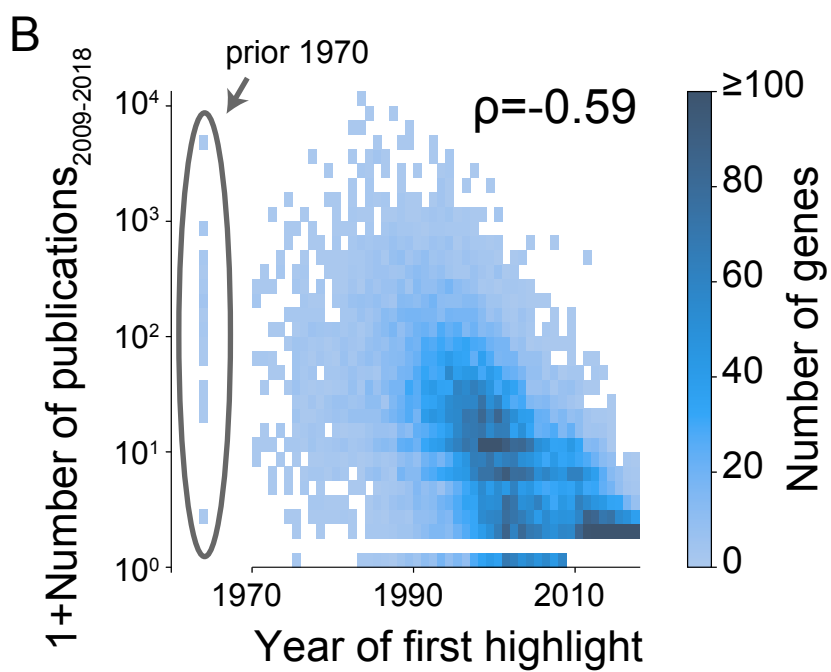

Supplement: S3 Fig — (A) Comparison of the number of articles highlighting a particular gene for 2009 to 2018 publications versus for 1980 to 2008 publications. (B) Comparison of the number of articles highlighting a particular gene for 2009 to 2018 publications versus year gene was first highlighted in a publication. Rho (ρ) indicates Spearman correlation coefficient. Combines data from MEDLINE, NCBI gene and taxonomy information, gene2pubmed, and PubTator. For data underlying the figure, see https://doi.org/10.21985/n2-b5bm-3b17. (PDF) [file pbio.3001520.s004.pdf]

Figure S4

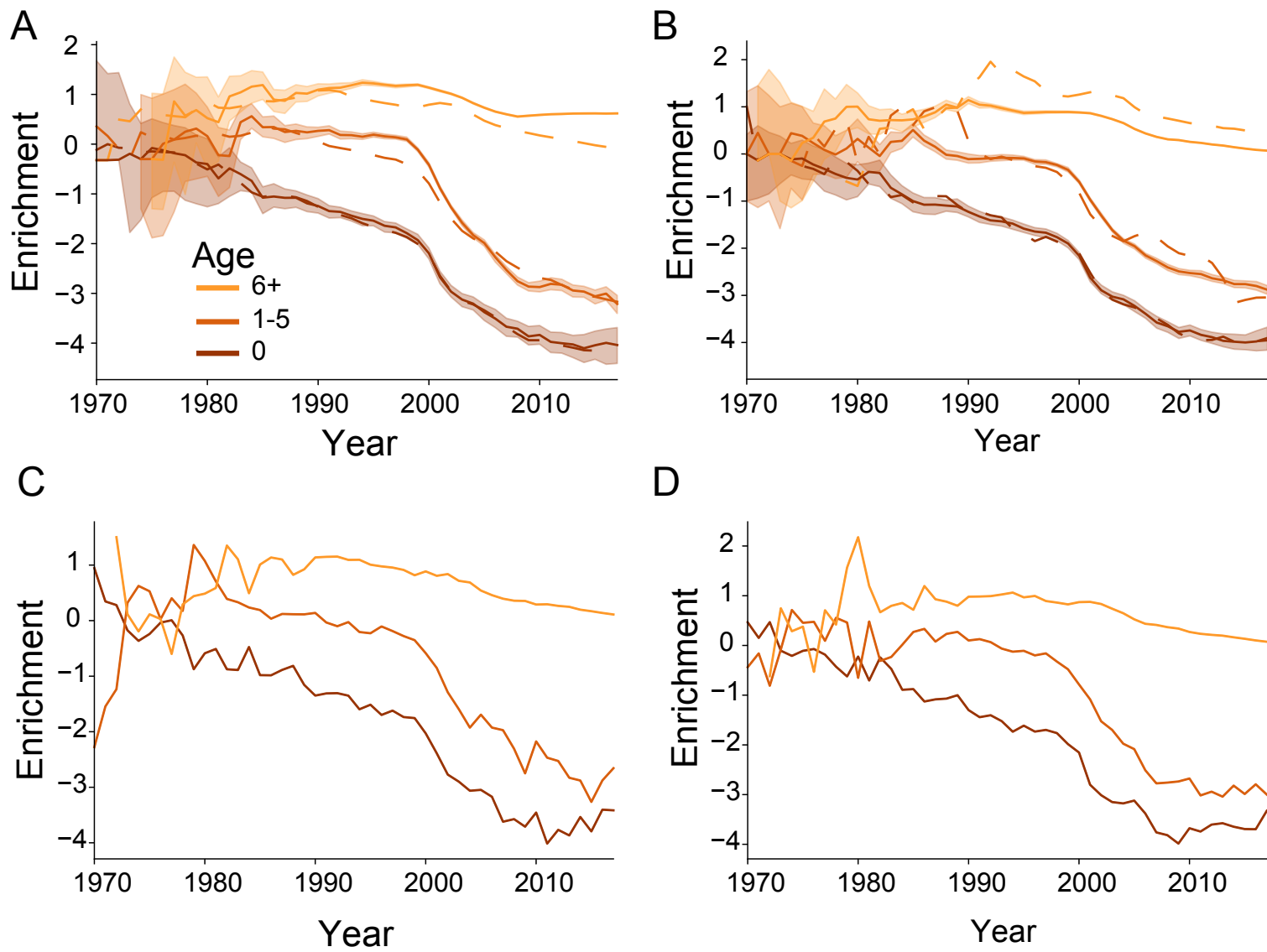

Supplement: S4 Fig — (A) Enrichment (log2 of ratio) of publications highlighting at least 1 loss-of-function intolerant gene (solid lines) aggregated by gene age and publication year (obtained as an average over a 3-year moving window). Loss-of-function intolerance was inferred from human polymorphisms. The solid lines show enrichment for loss-of-function intolerant genes, whereas for comparison the dashed lines show enrichment for genes that are not loss-of-function intolerant. Gene ages at publication are grouped identically in both panels A and B and as shown in legend of panel A. Error bars show 95% confidence intervals inferred by bootstrap. (B) As (A) but with loss-of-function intolerance inferred from the occurrence of non–wild-type phenotypes in systematic murine mutagenesis experiments. (C) Enrichment (log2 of ratio) of annual pairs of highlighted genes and research publications relative to the number of genes and the number of different phenotypes in a systematic murine mutagenesis experiment. (D) Enrichment (log2 of ratio) of annual pairs of highlighted genes and research publications relative to the number of genes and the number of different traits or disease in the NHGRI-EBI catalog of genome-wide association studies [34]. Note that C and D show 95% confidence intervals inferred by bootstrap of the genes but that bootstrap only has minimal effect on the enrichment. Combines data from MEDLINE, NCBI gene and taxonomy information, gene2pubmed, PubTator, Karczewski and colleagues [32], IMPC [33], and NHGRI-EBI GWAS catalog. For data underlying the figure, see https://doi.org/10.21985/n2-b5bm-3b17. (PDF) [file pbio.3001520.s005.pdf]

Figure S5

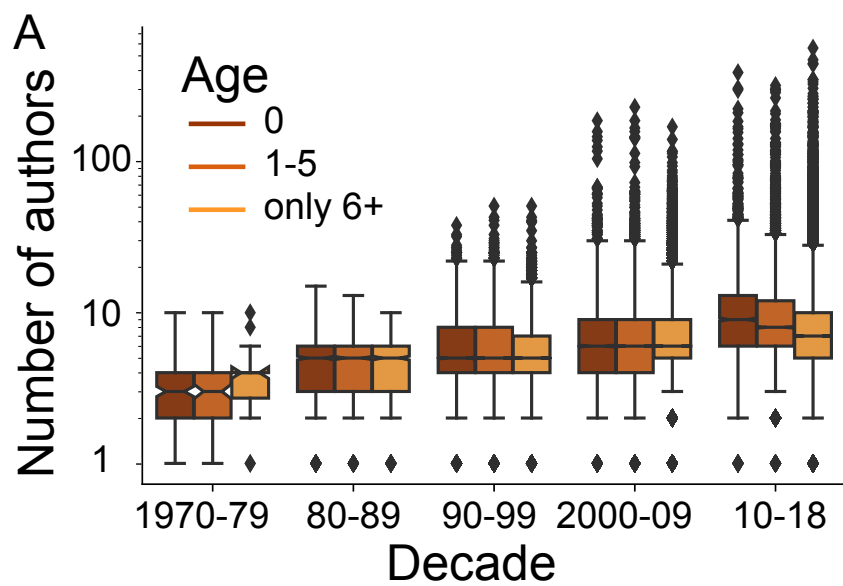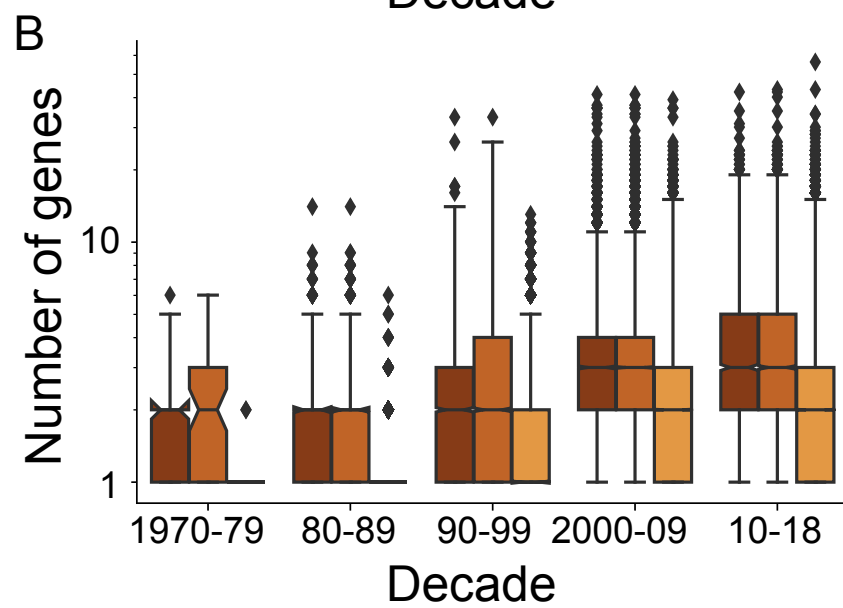

Supplement: S5 Fig — (A) Number of authors for publications highlighting at least 1 gene aggregated by gene age and publication decade. (B) Number of highlighted genes for publications highlighting at least 1 gene aggregated by gene age and publication decade. Notches in box plots indicate 95% confidence interval of the median. Gene ages at publication are grouped identically in both panels and as shown in legend of panel A. Combines data from MEDLINE, NCBI gene and taxonomy information, gene2pubmed, and PubTator. For data underlying the figure, see https://doi.org/10.21985/n2-b5bm-3b17. (PDF) [file pbio.3001520.s006.pdf]

Figure S6

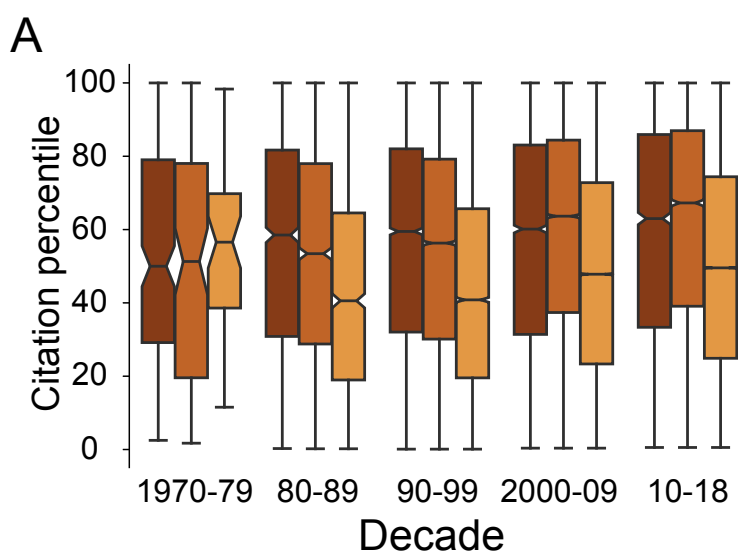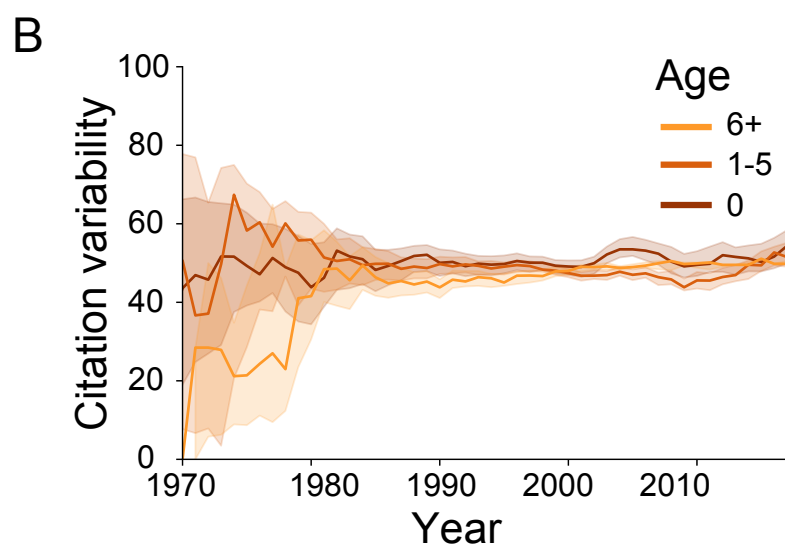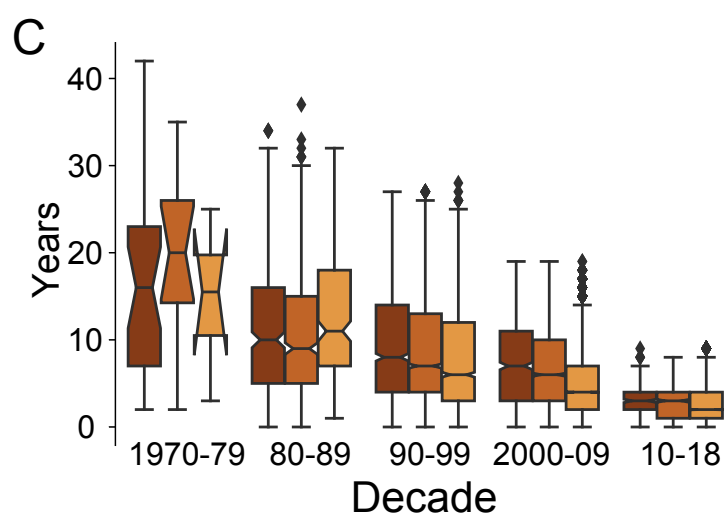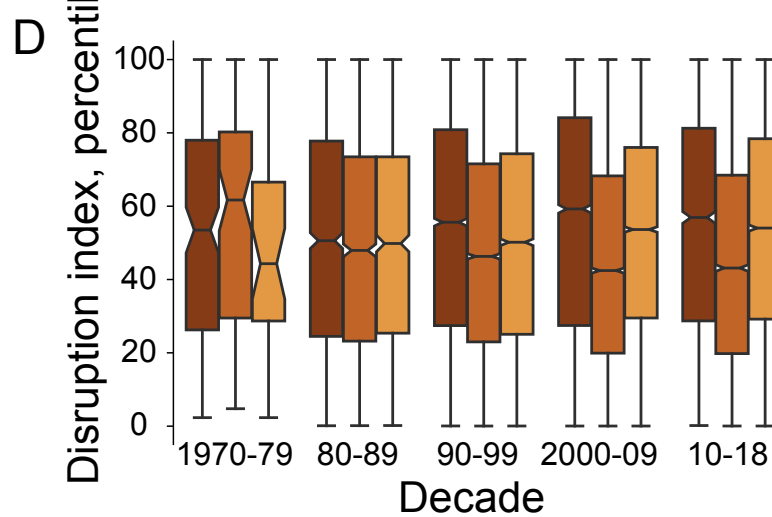

Supplement: S6 Fig — (A) Percentile of citations to publications highlighting at least 1 gene aggregated by age of highlighted gene and by decade. Notches indicate 95% confidence intervals of the median. (B) Citation variability as determined by the width of the interquartile range of the citations, normalized by percentiles of the year of the publication (with width being span between 25 and 75 percentiles). Shaded area indicates 95% confidence intervals inferred by bootstrap. Year indicates center of a 3-year sliding window used for analysis. (C) Years until first citation by a clinical trial to a publication highlighting at least 1 gene aggregated by age of highlighted gene and by decade. (D) Percentile of disruption index of a publication highlighting at least 1 gene aggregated by age of highlighted gene and by decade. Brown indicates publications with at least 1 gene that has not been highlighted in any preceding year. Dark orange indicates publications with at least 1 gene that was first highlighted during the 5 preceding years. Light orange indicates publications that only highlight genes that have been first highlighted 6 or more years earlier. Combines data from MEDLINE, NCBI gene and taxonomy information, gene2pubmed, PubTator and iCite. For data underlying the figure, see https://doi.org/10.21985/n2-b5bm-3b17. (PDF) [file pbio.3001520.s007.pdf]

Figure S7

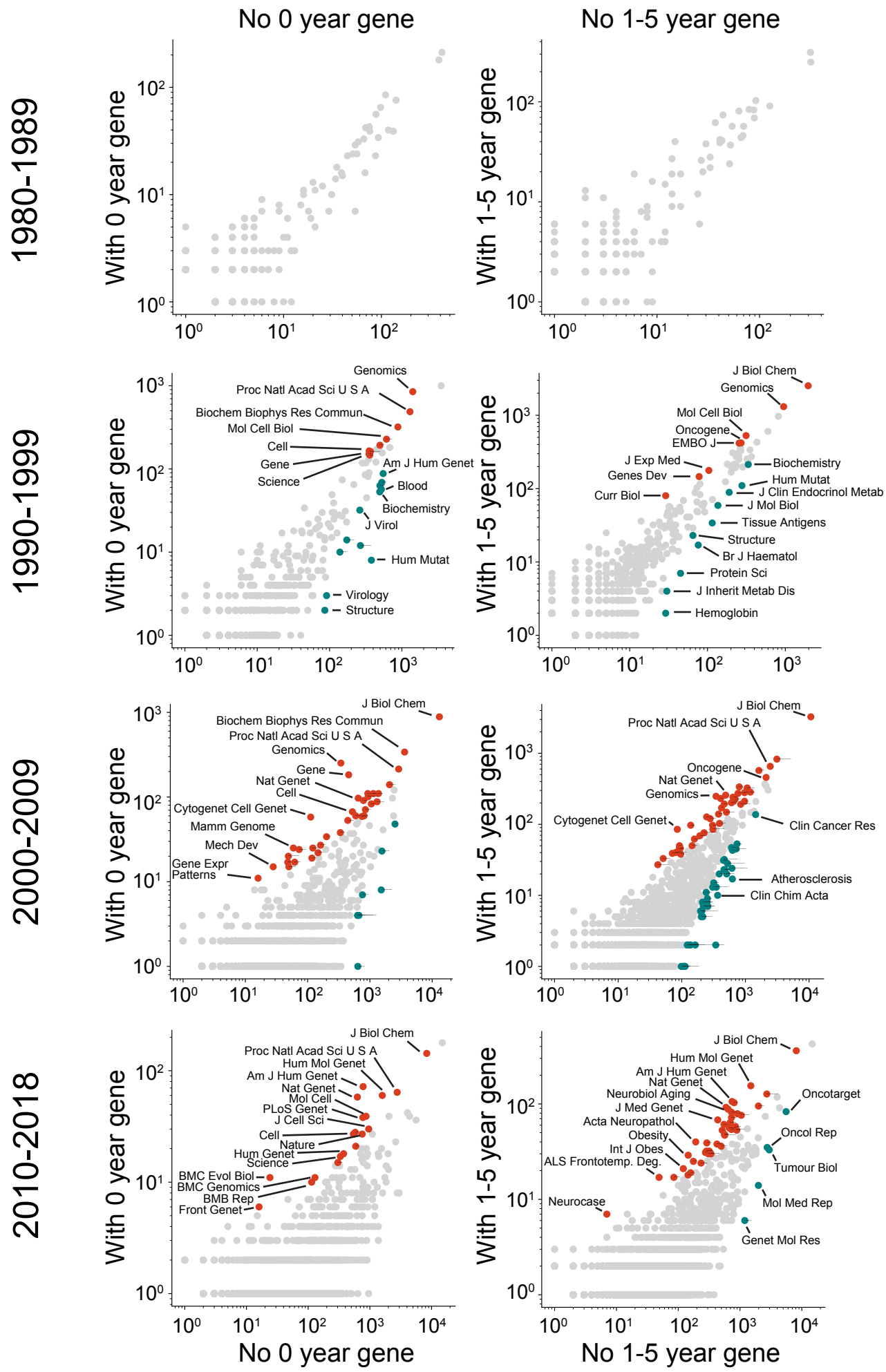

Supplement: S7 Fig — Plots within the left column focus on publications that highlight at least 1 new gene that had not been highlighted in any preceding year (0-year genes), whereas plots within the right column focus on publications that highlight at least 1 gene that was recently highlighted for the first time (1- to 5-year genes). In each plot, we compare the number of publications in a given journal highlighting at least 1 new or recent gene target against the total number of other publications in the same journal. Each circle represents an individual journal that published at least 1 study highlighting at least 1 gene. Red (cyan) circles indicate journals significantly enriched (depleted) for respective early-stage research. Combines data from MEDLINE, NCBI gene and taxonomy information, gene2pubmed, and PubTator. For data underlying the figure, see https://doi.org/10.21985/n2-b5bm-3b17. (PDF) [file pbio.3001520.s008.pdf]

Figure S8

1980-1989

No 0 year gene

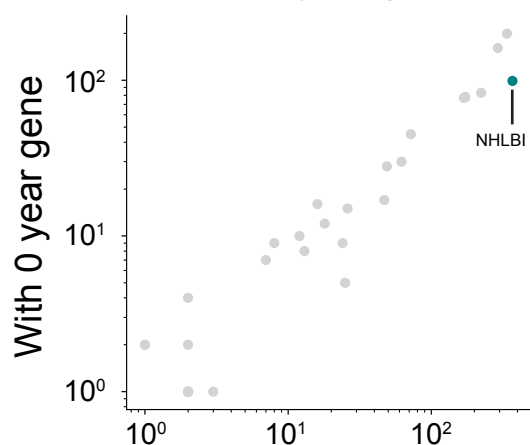

No 1-5 year gene

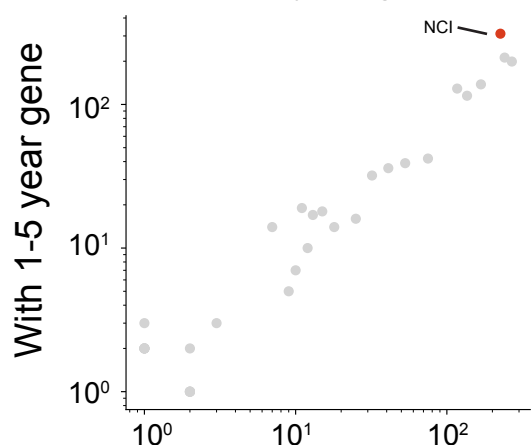

1990-1999

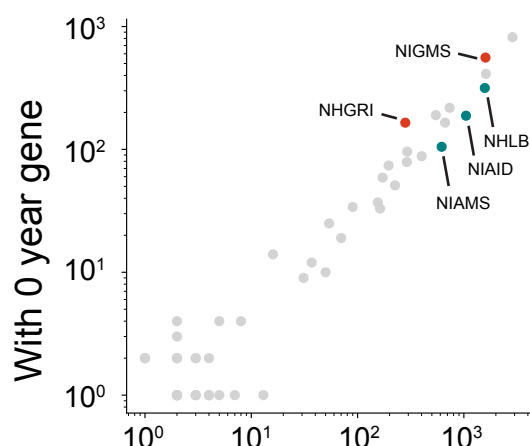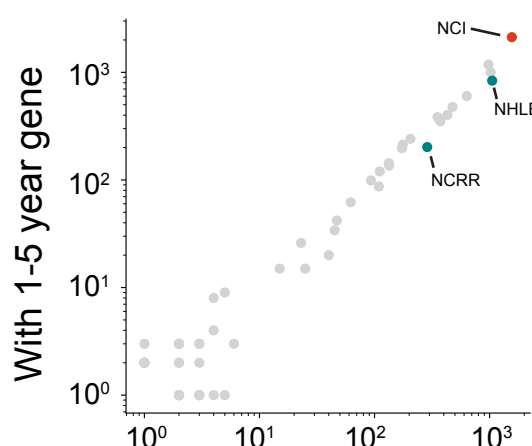

2000-2009

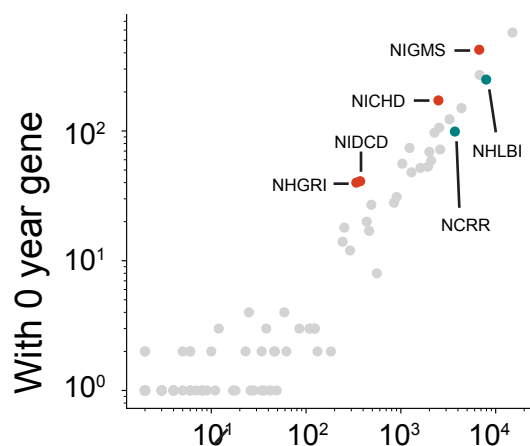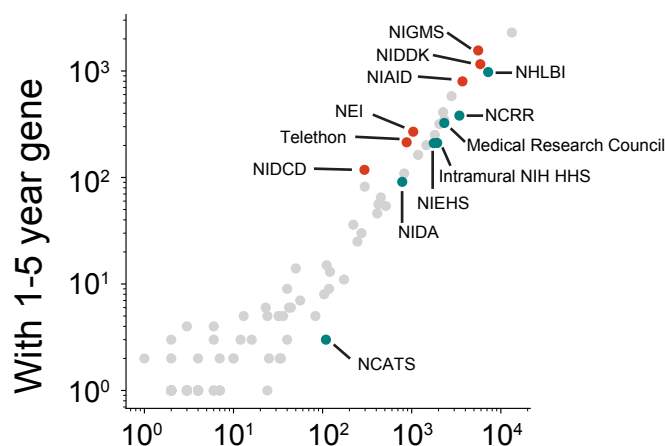

2010-2018

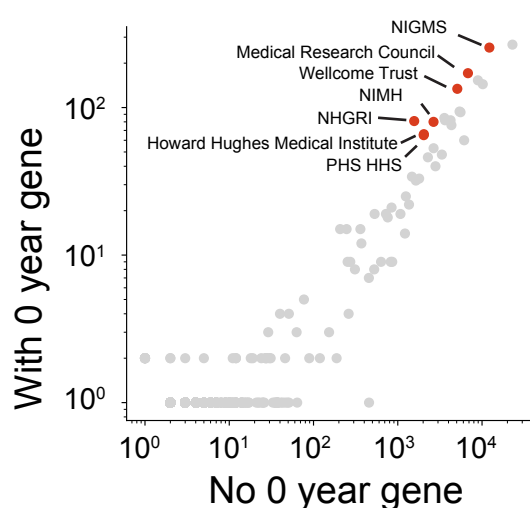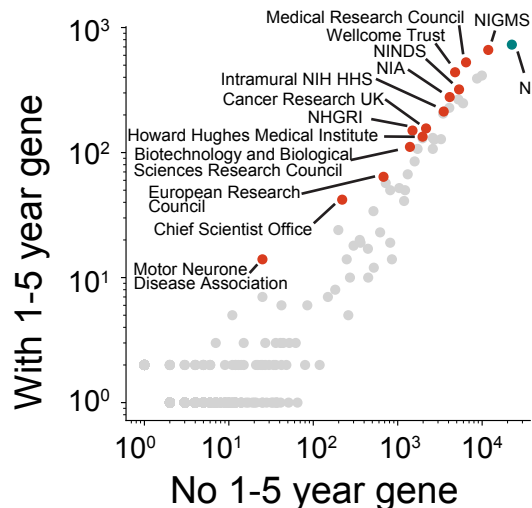

Supplement: S8 Fig — Plots within the left column focus on publications that highlight at least 1 new gene that had not been highlighted in any preceding year (0-year genes), whereas plots within the right column focus on publications that highlight at least 1 gene that was recently highlighted for the first time (1- to 5-year genes). In each plot, we compare the number of publications acknowledging funding from a given agency highlighting at least 1 new or recent gene against the total number of other publications acknowledging funding from the same agency. Each circle represents a funding agency acknowledged in a least 1 publication that highlighting at least 1 gene. Red (cyan) circles indicate funding agency significantly enriched (depleted) for respective early-stage research. Combines data from MEDLINE, NCBI gene and taxonomy information, gene2pubmed, PubTator, and ExPORTER. (PDF) [file pbio.3001520.s009.pdf]

Figure S9

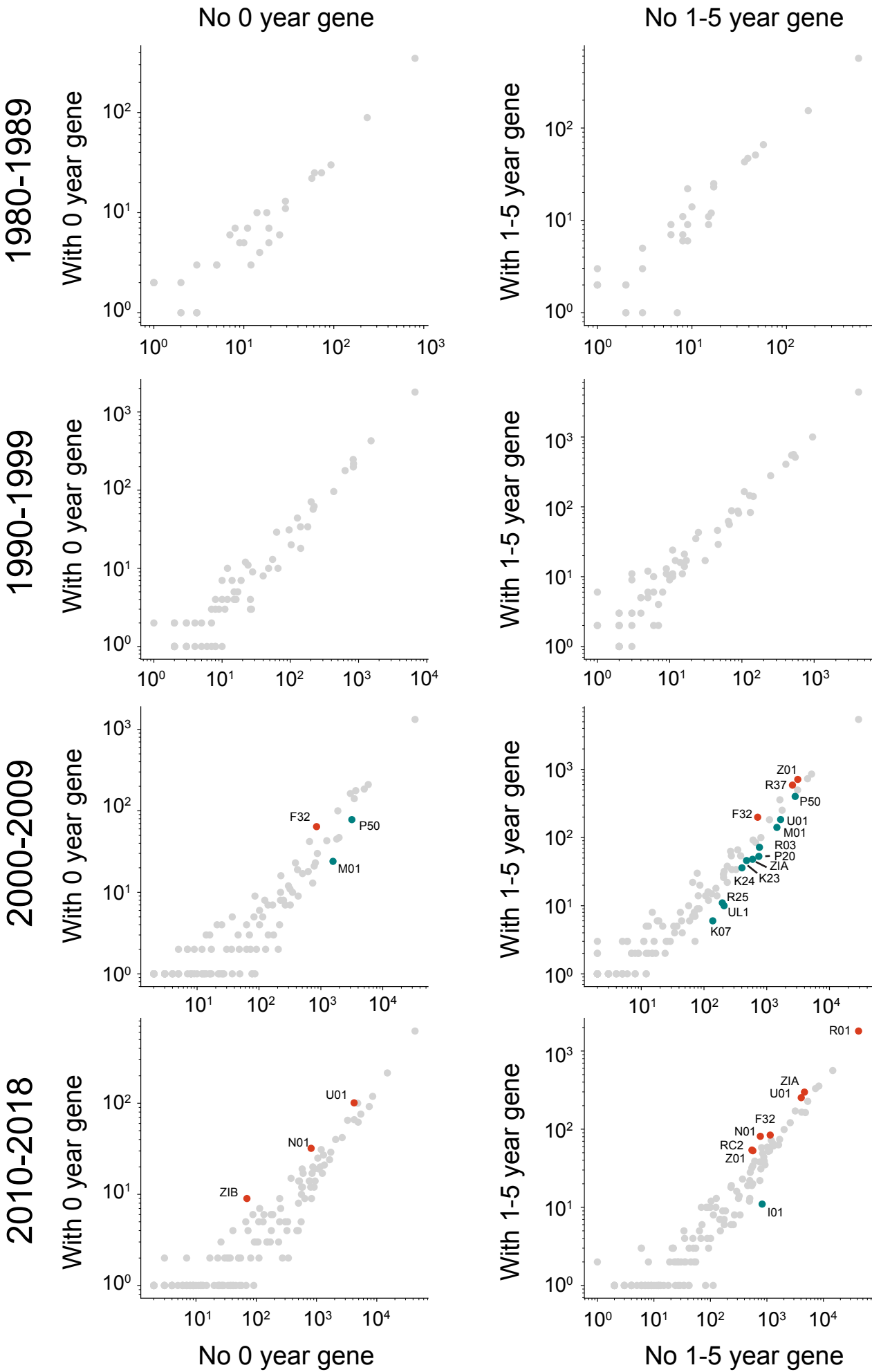

Supplement: S9 Fig — Plots within the left column focus on publications that highlight at least 1 new gene that had not been highlighted in any preceding year (0-year genes), whereas plots within the right column focus on publications that highlight at least 1 gene that was recently highlighted for the first time (1- to 5-year genes). In each plot, we compare the number of publications acknowledging funding from a given activity code highlighting at least 1 new or recent gene against the total number of other publications acknowledging funding from the same activity. Each circle represents an activity code for NIH funding acknowledged in a least 1 publication that highlighting at least 1 gene. Red (cyan) circles indicate activity codes significantly enriched (depleted) for respective early-stage research. Combines data from MEDLINE, NCBI gene and taxonomy information, gene2pubmed, and PubTator. For data underlying the figure, see https://doi.org/10.21985/n2-b5bm-3b17. NIH, National Institutes of Health. (PDF) [file pbio.3001520.s010.pdf]

Figure S10

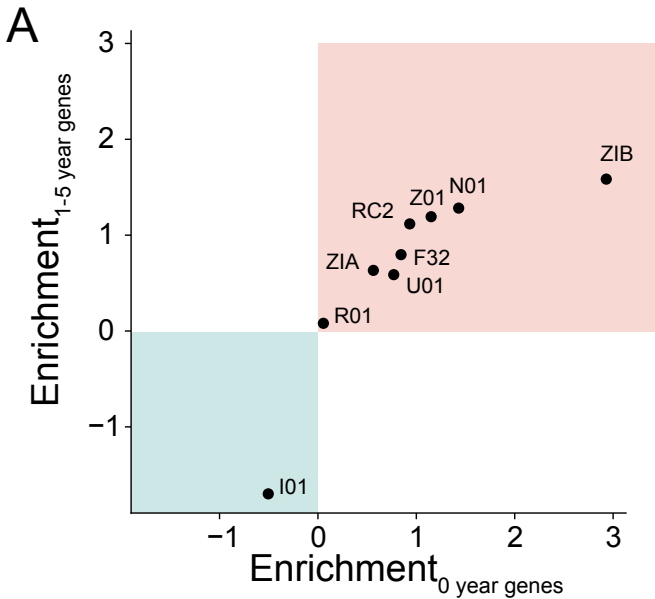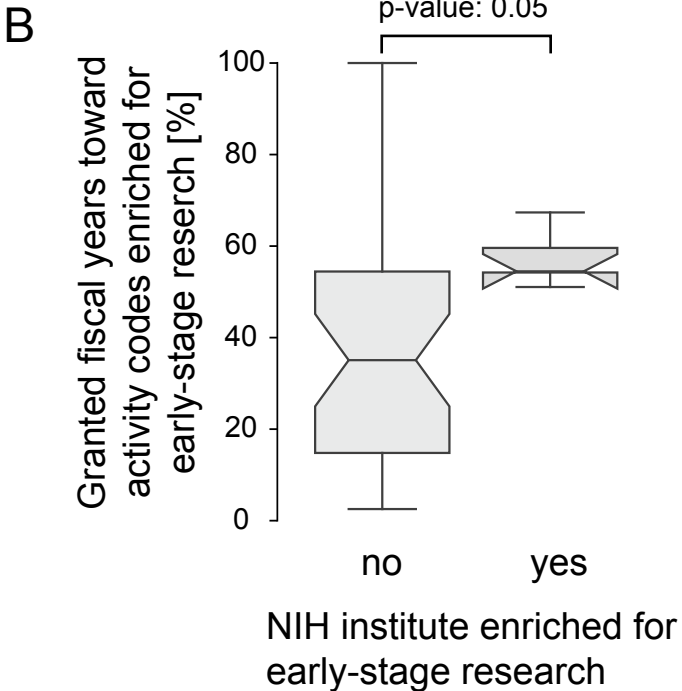

Supplement: S10 Fig — (A) Comparison of fold enrichment (log2 of ratio) for activity codes from NIH funding that are significantly enriched or depleted for new gene targets or recent gene targets. Note that for any given activity code, the enrichment may not be statistically significant for both axes. (B) Box plot showing share of grants—measured through fiscal years (as grant durations can vary among activity codes)—that go toward enriched activity codes between 2010 and 2018 (salmon box of panel A) for NIH institutes that do not (no) or do (yes) enrich for early-stage research. p-Value is obtained by 2-sided Mann–Whitney U test. Combines data from MEDLINE, NCBI gene and taxonomy information, gene2pubmed, PubTator, and ExPORTER. For data underlying the figure, see https://doi.org/10.21985/n2-b5bm-3b17. NIH, National Institutes of Health. (PDF) [file pbio.3001520.s011.pdf]

Figure S11

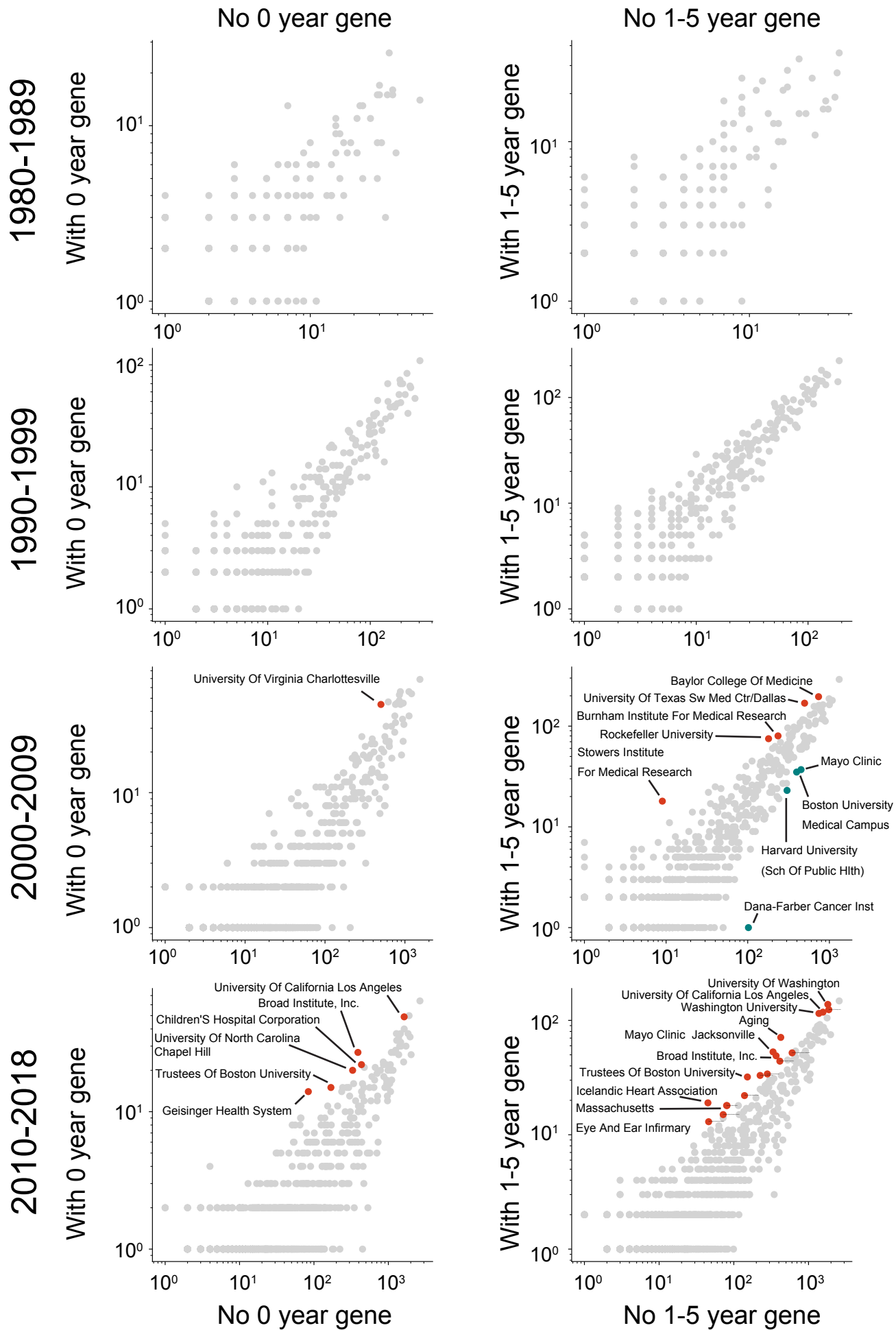

Supplement: S11 Fig — Plots within the left column focus on publications that highlight at least 1 new gene that had not been highlighted in any preceding year (0-year genes), whereas plots within the right column focus on publications that highlight at least 1 gene that was recently highlighted for the first time (1- to 5-year genes). In each plot, we compare the number of publications with at least 1 author affiliated with a given institution highlighting at least 1 new or recent gene against the total number of other publications with at least 1 author affiliated with the same institution. Each circle represents an institution associated with at least 1 publication that highlighting at least 1 gene. Red (cyan) circles indicate institutions significantly enriched (depleted) for respective early-stage research. Combines data from MEDLINE, NCBI gene and taxonomy information, gene2pubmed, PubTator, and ExPORTER. For data underlying the figure, see https://doi.org/10.21985/n2-b5bm-3b17. (PDF) [file pbio.3001520.s012.pdf]

Figure S12

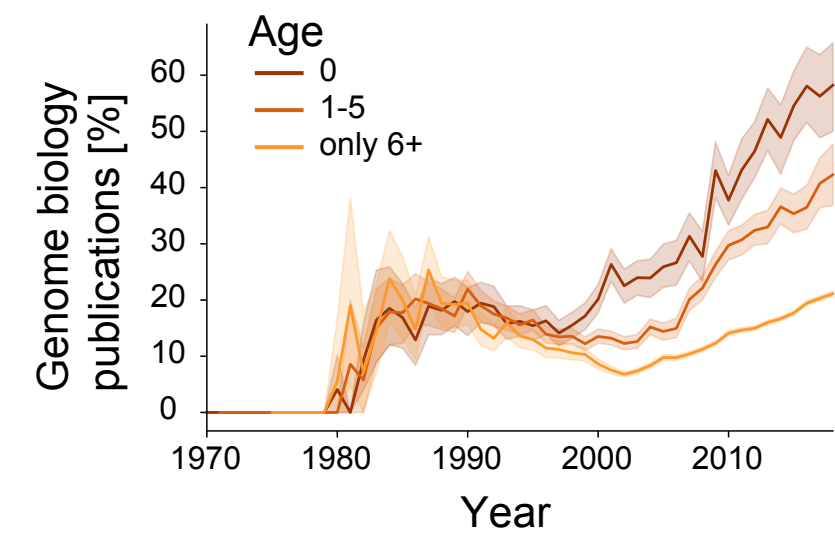

Supplement: S12 Fig — Shaded area indicates 95% confidence intervals inferred by bootstrap. Combines data from MEDLINE, NCBI gene and taxonomy information, gene2pubmed, and PubTator. For data underlying the figure, see https://doi.org/10.21985/n2-b5bm-3b17. (PDF) [file pbio.3001520.s013.pdf]

Figure S13

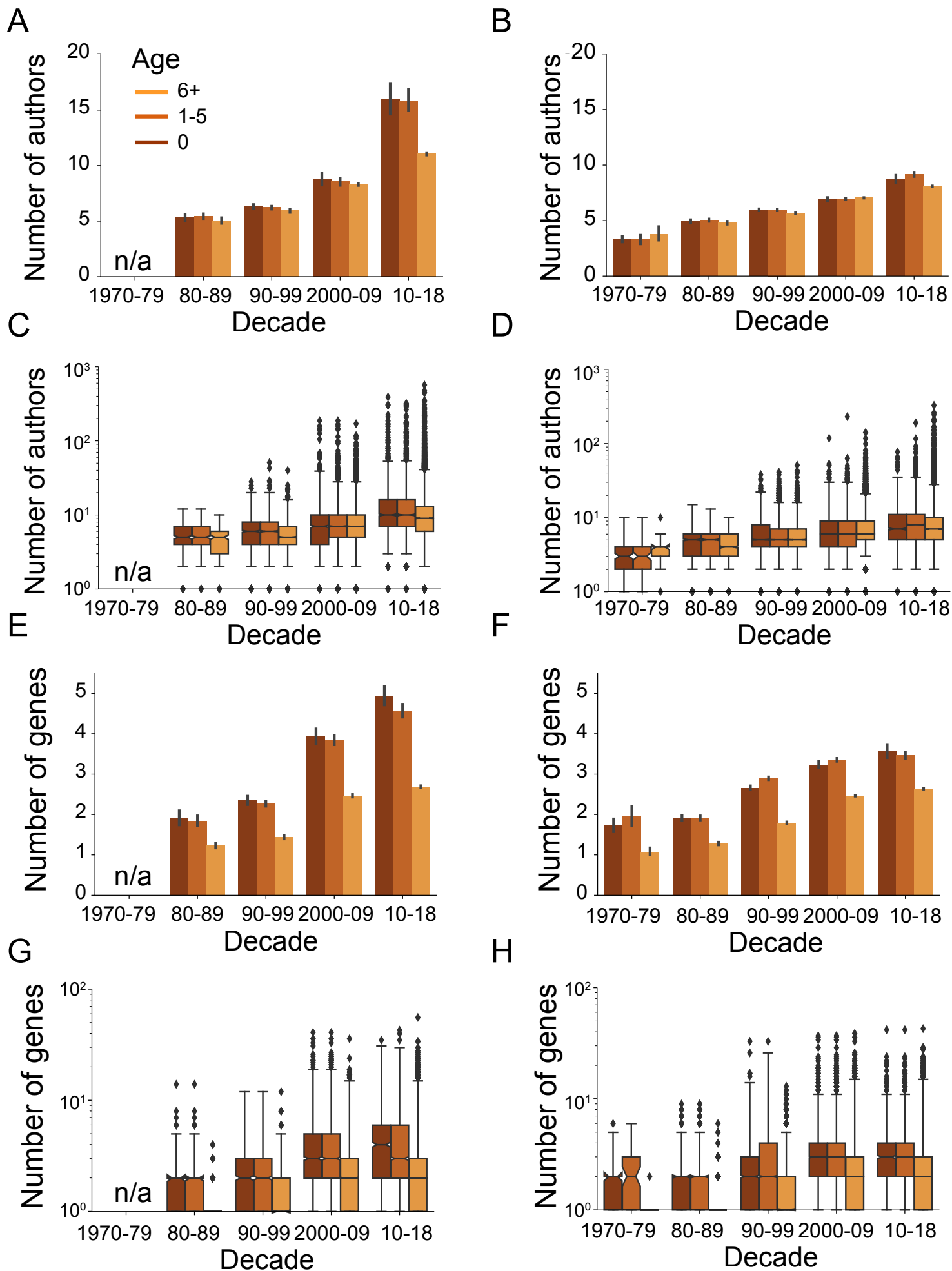

Supplement: S13 Fig — (A) Mean number of authors for publications categorized as genome biology highlighting at least 1 gene aggregated by age of highlighted gene and by decade. (B) Mean number of authors for publications not categorized as genome biology highlighting at least 1 gene aggregated by age of highlighted gene and by decade. (C) Box plot of number of authors for publications categorized as genome biology highlighting at least 1 gene aggregated by age of highlighted gene and by decade. (D) Box plot of number of authors for publications not categorized as genome biology highlighting at least 1 gene aggregated by age of highlighted gene and by decade. (E) Mean number of highlighted genes for publications categorized as genome biology highlighting at least 1 gene aggregated by age of highlighted gene and by decade. (F) Mean number of highlighted genes for publications not categorized as genome biology highlighting at least 1 gene aggregated by age of highlighted gene and by decade. (G) Box plot of number of highlighted genes for publications categorized as genome biology highlighting at least 1 gene aggregated by age of highlighted gene and by decade. (H) Box plot of number of highlighted genes for publications not categorized as genome biology highlighting at least 1 gene aggregated by age of highlighted gene and by decade. Error bars show 95% confidence intervals inferred by bootstrap. Gene ages at publication are grouped identically in all panels and as shown in legend of panel A. Notches in box plots indicate 95% confidence intervals of the median. n/a indicates non applicability due to absence of genome biology publications in 1970 to 1979. Combines data from MEDLINE, NCBI gene and taxonomy information, gene2pubmed, and PubTator. For data underlying the figure, see https://doi.org/10.21985/n2-b5bm-3b17. (PDF) [file pbio.3001520.s014.pdf]

Figure S14

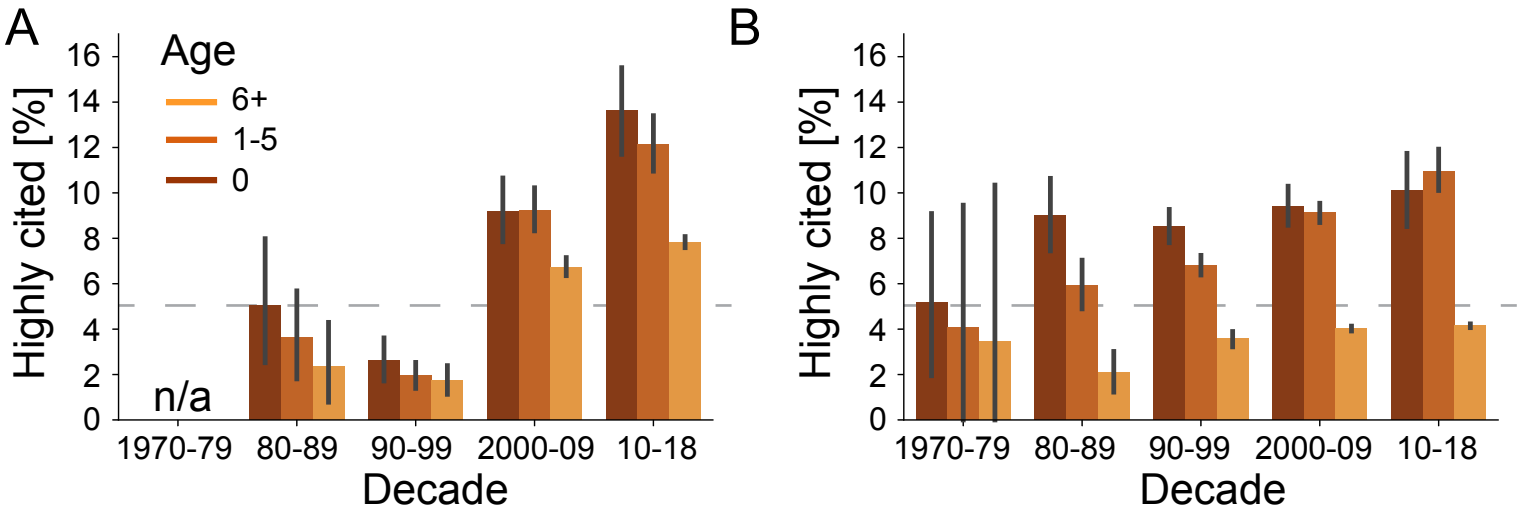

Supplement: S14 Fig — (A) Percentage of highly cited publications categorized in genome biology (among top 5% of indicated year) aggregated by decade and age of highlighted gene. (B) Percentage of highly cited publications not categorized in genome biology (among top 5% of publication year) aggregated by decade and age of highlighted gene. Dashed line shows 5% baseline. Gene ages at publication are grouped identically in all panels and as shown in legend of panel A. n/a indicates non applicability due to absence of genome biology publications in 1970 to 1979. Combines data from MEDLINE, NCBI gene and taxonomy information, gene2pubmed, PubTator, and iCite. For data underlying the figure, see https://doi.org/10.21985/n2-b5bm-3b17. (PDF) [file pbio.3001520.s015.pdf]

Figure S15

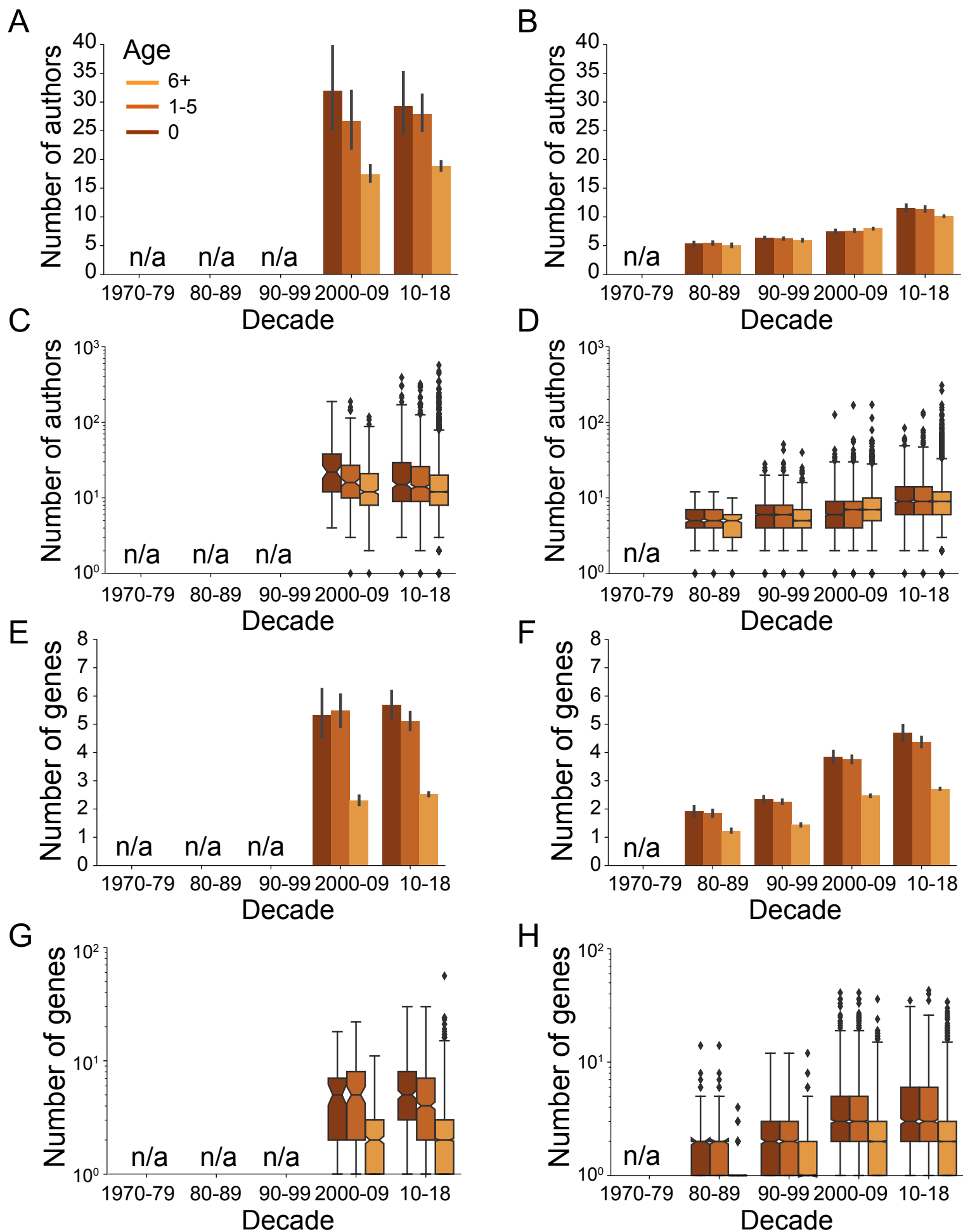

Supplement: S15 Fig — (A) Mean number of authors for publications categorized as genome-wide association study highlighting at least 1 gene aggregated by age of highlighted gene and by decade. (B) Mean number of authors for publications categorized as not being a genome-wide association study highlighting at least 1 gene aggregated by age of highlighted gene and by decade. (C) Box plot of number of authors for publications categorized as genome-wide association study highlighting at least 1 gene aggregated by age of highlighted gene and by decade. (D) Box plot of number of authors for publications categorized as not being a genome-wide association study highlighting at least 1 gene aggregated by age of highlighted gene and by decade. (E) Mean number of highlighted genes for publications categorized as genome-wide association study highlighting at least 1 gene aggregated by age of highlighted gene and by decade. (F) Mean number of highlighted genes for publications categorized as not being a genome-wide association study highlighting at least 1 gene aggregated by age of highlighted gene and by decade. (G) Box plot of number of highlighted genes for publications categorized as genome biology and genome-wide association study highlighting at least 1 gene aggregated by age of highlighted gene and by decade. (H) Box plot of number of highlighted genes for publications categorized as not being a genome-wide association study highlighting at least 1 gene aggregated by age of highlighted gene and by decade. Error bars show 95% confidence intervals inferred by bootstrap. Gene ages at publication are grouped identically in all panels and as shown in legend of panel A. Notches in box plots indicate 95% confidence intervals of the median. n/a indicates non applicability due to absence of genome biology publications in 1970 to 1979 and genome-wide association studies in 1970 to 1999. Combines data from MEDLINE, NCBI gene and taxonomy information, gene2pubmed, and PubTator. For data underlying the fig [file pbio.3001520.s016.pdf]

Figure S16

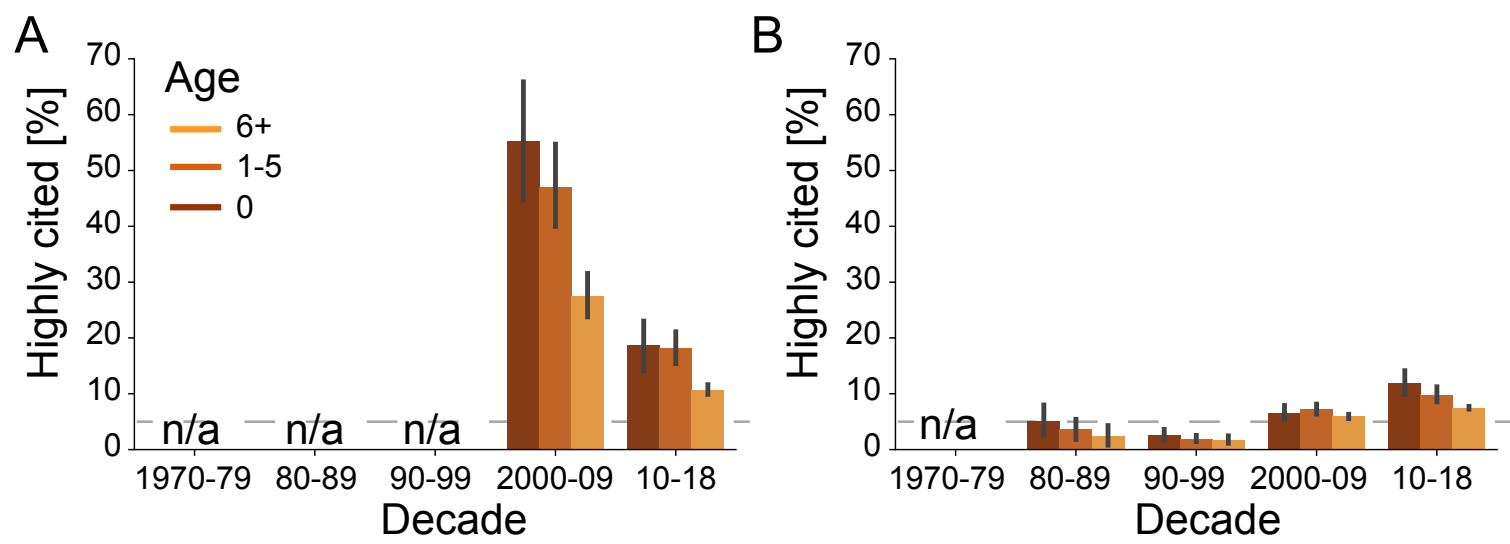

Supplement: S16 Fig — (A) Percentage of highly cited publications (among top 5% of indicated year) for publications categorized as genome-wide association study aggregated by decade and age of highlighted gene. (B) Percentage of highly cited (among top 5% of publication year) publications for publications not categorized as not being a genome-wide association study aggregated by decade and age of highlighted gene. Dashed line shows 5% baseline. Gene ages at publication are grouped identically in all panels and as shown in legend of panel A. n/a indicates non applicability due to absence of genome biology publications in 1970 to 1979 and genome-wide association studies in 1970 to 1999. Combines data from MEDLINE, NCBI gene and taxonomy information, gene2pubmed, PubTator, and iCite. For data underlying the figure, see https://doi.org/10.21985/n2-b5bm-3b17. (PDF) [file pbio.3001520.s017.pdf]

Figure S17

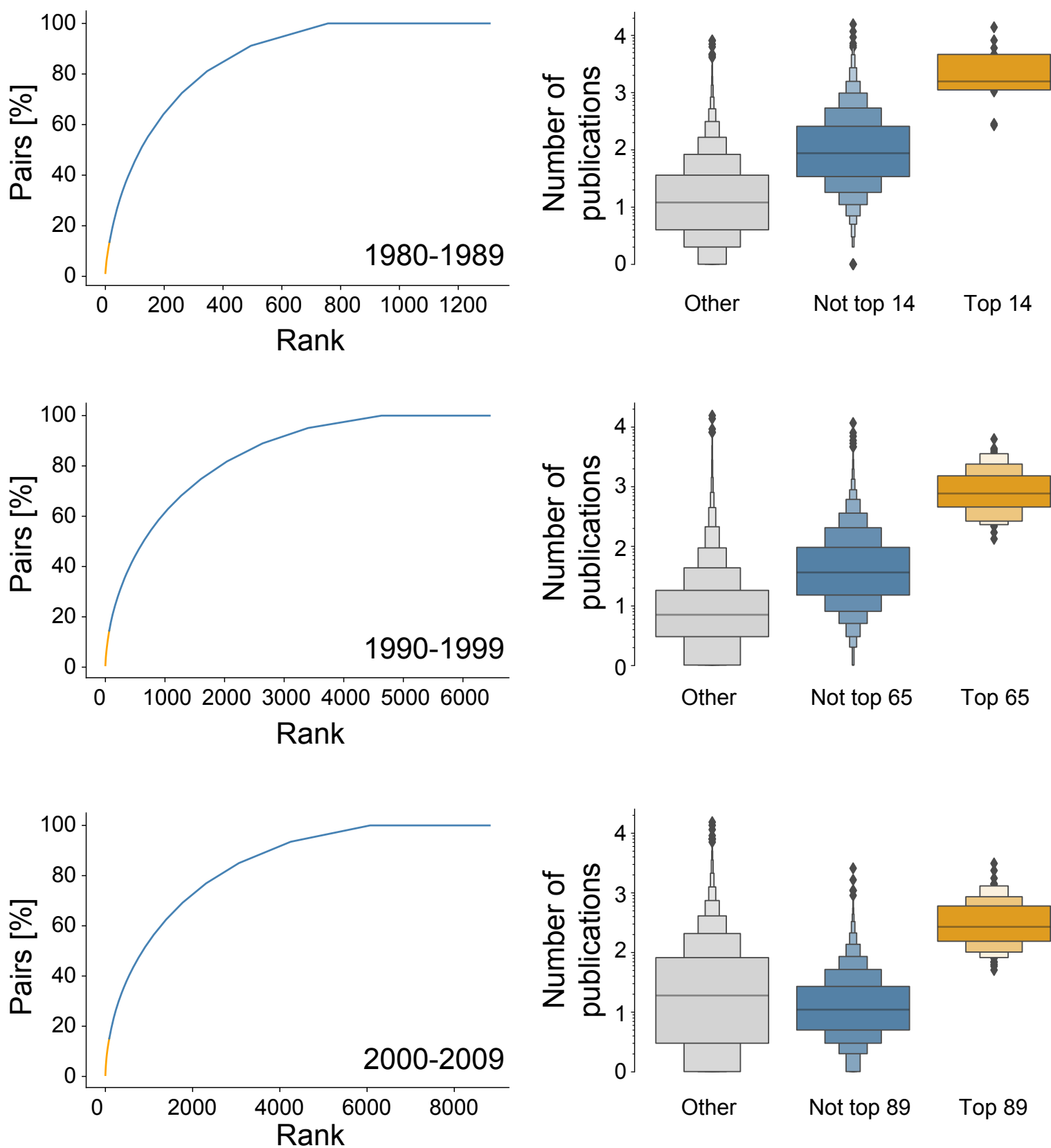

Supplement: S17 Fig — Note that the number of genes in the top 1% increases over time because so does the total number of gene highlighted in each period. (Left) Cumulative distribution of pairs of highlighted genes and publications in considered period highlighting recent gene targets (first highlighted 1 to 5 years before). The x-axis ranks genes by number of publications highlighting them, with rank 0 corresponding to the gene highlighted in the most publications within a given decade. The y-axis tallies the percentage of all pairs of highlighted genes in publications in considered period highlighting recent gene targets with a lower or equal rank. We emphasize in orange the top 1% genes which together account for over 15% of all pairs. (Right) Letter plots of all highlighted genes reporting for each gene the total number of publications until 2018 for genes that still were a recent target during the time period of the respective left panel with top 1% genes in orange and other 99% gene in orange. For comparison, all other genes (which are no recent gene target during time period) are shown in gray. Area of boxes indicates share of values, with heights of boxes following letter style dimensions—innermost boxes contain 25 to 75 percentiles of values and subsequent boxes 12.75 to 87.5 percentiles of values, etc. Combines data from MEDLINE, NCBI gene and taxonomy information, gene2pubmed, and PubTator. For data underlying the figure, see https://doi.org/10.21985/n2-b5bm-3b17. (PDF) [file pbio.3001520.s018.pdf]
