# Supplementary material for: The characteristics of early-stage research into human genes are substantially different from subsequent research
Source: PLoS Biol. 2022 Jan 6;20(1):e3001520. doi: 10.1371/journal.pbio.3001520 (PMC8769369; doi:10.1371/journal.pbio.3001520)
Supplement: S1 PRISMA Flow Diagram — (DOCX) [file pbio.3001520.s019.docx]

**Identification of studies via databases and registers**

Records removed *before screening*:

Duplicate records removed (n = 18)

Records marked as ineligible by automation tools (n = 0)

Records removed for other reasons (n = 0)

Records identified from*:

Databases (n = 30,419,065)

Registers (n = 0)

**Identification**

Records excluded**

(n = 0)

Records screened

(n = 30,419,047)

Reports sought for retrieval

(n = 30,419,047)

Reports not retrieved

(n = 0)

**Screening**

Reports excluded:

Published after 2018 (n=1,334,568)

Not research article (n = 4,881,536)

Not about human genes according to gene2pubmed (n = 23,609,051)

No human protein-coding gene in title or abstract according to text mining via pubtator (n = 108,092)

Reports assessed for eligibility

(n = 30,419,047)

Studies included in review

(n = 485,800)

Reports of included studies

(n = 485,800)

**Included**

*Consider, if feasible to do so, reporting the number of records identified from each database or register searched (rather than the total number across all databases/registers).

**If automation tools were used, indicate how many records were excluded by a human and how many were excluded by automation tools.

*From:*  Page MJ, McKenzie JE, Bossuyt PM, Boutron I, Hoffmann TC, Mulrow CD, et al. The PRISMA 2020 statement: an updated guideline for reporting systematic reviews. BMJ 2021;372:n71. doi: 10.1136/bmj.n71

For more information, visit: <http://www.prisma-statement.org/>
