# Supplementary material for: The characteristics of early-stage research into human genes are substantially different from subsequent research
Source: PLoS Biol. 2022 Jan 6;20(1):e3001520. doi: 10.1371/journal.pbio.3001520 (PMC8769369; doi:10.1371/journal.pbio.3001520)
Supplement: Attachment — Submitted filename: reviewer_comments.docx [file pbio.3001520.s020.docx]

Reviewer #1:

In this paper, Stoeger and Amaral analyze the contexts that have historically enabled early-stage research into human genes to better understand how such research can be sustained and revitalized in the contemporary research ecosystem. I have a few comments/questions about the overall framing of the manuscript and how much these results might be driven by GWAS publications in the last ~15 years, but overall I think it is a thorough and interesting contribution to the metaresearch literature, and should spark some healthy debate about balancing depth and breadth in genetics/genomics research and the surrounding incentive structures.

We thank the reviewer for the kind words and have addressed below concerns.

Major comments:
1. The paragraph in lines 38-49 is extremely unclear to me. The authors state that the narrow focus on well-studied genes raises "significant concerns" about knowledge generation. The examples the authors cite to motivate this claim, however, seem quite anecdotal and/or poorly communicated. First, the sentence in lines 39-41 (with reference to Ioannidis, 2005) seems to be insinuating that results reported for well-studied genes are increasingly enriched for false positives, relative to the results reported for understudied genes. Is there any evidence that the false positive *rate* correlates with a gene's popularity in the literature? This would indeed be troubling if true, but it seems quite speculative. Also, the authors suggest that incorrect research results are selectively accumulating among already well-studied genes, but the claim that this is a "significant concern" overlooks a lot of important context--perhaps such incorrect results are primarily appearing in low-impact or predatory journals (e.g., Genet Mol Res, as described in lines 270-272) or are enriched in publications from a small subset of authors with poor data or incorrect assumptions. Regardless, this claim feels too strong without some empirical contrast showing that the false positive rate is higher for well-studied genes and/or those false positives are qualitatively more detrimental to scientific progress than false positive results pertaining to understudied genes.

We agree with the reviewer that our initial submission leaves important points unclear. For this reason, we delete the paragraph identified by the reviewer. We now address the practical risks of a narrow focus in a new paragraph (lines 134-144).

Note also that the association between research popularity and false claims is the topic of an ongoing collaboration with Jennifer Byrne. For potential curiosity of the reviewer, we advertise to the following preprint of ours <https://www.biorxiv.org/content/10.1101/2021.07.29.453321v1>, which suggests that wrong claims could be particularly challenging for computational approaches that infer knowledge from the literature. We will present the association between research popularity and wrong claims in future work.

Second, I did not track how the GSK study relates to this concern--the study found that published claims about well-studied genes were not especially predictive of clinical trial success, but this only seems to be of concern if there is some contrast showing that published claims of understudied genes are consistently more accurate (ref 17 also seems out of place here, as it is an opinion piece musing about the need for more stringent significance thresholds in medical research and doesn't contribute any empirical evidence supporting the claims made in line 44).

We thank the reviewer for this pointed observation. We are particularly attuned to the fact that the reviewer’s comment touches upon the interesting issue of how machine and human readers of scientific literature are distinctly affected by annotation biases and the traditions and heuristics typically used to counter them.

To sharpen the focus of the manuscript, we chose to simply deleting the paragraph and replacing it by a paragraph that that uses two more specific examples: First, on literature on functional enrichment analyses that shows that absent knowledge could lead to false-negative findings in subsequent analyses. Second, that an uneven number of studies can shape inferred protein-protein interaction networks in manners that do not resemble protein-protein interaction networks without these biases. (lines 134-144).

In addition, we rephrased a sentence in the study limitations section, which also referred to the GSK study. It now no longer provides a comparative judgment.

Specifically: “such a view might be myopic as independent re-evaluations of the data underlying research articles can be a valid and successful research strategy [reference to GSK].”

2. Lines 168-169: It seems likely that many of the recent early-stage publications produced by larger teams are coming from GWAS/TWAS studies where teams are more or less agnostically searching for association signals in any and all genes. This shouldn't be particularly surprising, since the number of authors correlates with GWAS sample size and thus also correlates with power to detect novel associations, which presumably yields additional hits in relatively understudied genes. To me, this suggests the most parsimonious answer to "how do we encourage early-stage research on understudied genes?" is simply "fund/collaborate on bigger GWAS!", which seems somewhat antithetical to the authors' goal of increasing innovation in the life sciences.

See also lines 189-192—"since the mid-2000s" aligns with when the first successful GWAS were published, so I'd expect much of what the authors are interpreting as recent bibliometric recognition/rewards for early-stage research could alternatively be interpreted as bibliometric recognition for GWAS as a methodology and subdiscipline of genomics (this is even alluded to in lines 333-337, where the NIH-funded organizations with the strongest enrichment for early-stage research are solely driven by GWAS-related publications, and lines 416-417, describing the initial discoveries of FTO and C9orf72 via GWAS). 

Ultimately, I'm curious if these results and interpretations change when GWAS publications are excluded from the analyses. Struck et al. (2018) [[https://urldefense.com/v3/__https://www.ncbi.nlm.nih.gov/pmc/articles/PMC6090631/__;!!Dq0X2DkFhyF93HkjWTBQKhk!Gf4eJAg3_wL_lSYqGL7CRwLdCtyHzN1tls-M6yZHQFcyF03Ja9U9mKu3yviykZ_6dx0ohJ6z6Fg$](https://urldefense.com/v3/__https:/www.ncbi.nlm.nih.gov/pmc/articles/PMC6090631/__;!!Dq0X2DkFhyF93HkjWTBQKhk!Gf4eJAg3_wL_lSYqGL7CRwLdCtyHzN1tls-M6yZHQFcyF03Ja9U9mKu3yviykZ_6dx0ohJ6z6Fg$) ] is an essential reference here, as they show that genes newly implicated by GWAS in complex disease do experience additional publications compared to control genes with similar publication histories. (The trend described in lines 400-420 was previously characterized by Struck et al., where a subset of recently-highlighted genes discovered via GWAS have received extraordinary attention in the literature in a short period of time).

This comment raises an important possibility that was not fully addressed by us.

We now single out GWAS/TWAS among genome research and find that our also outside of GWAS/TWAS genome research the bibliometric trends described by us hold.

Nonetheless, the same split analysis demonstrates that GWAS/TWAS have more authors, address more genes, and are more highly cited.

We revised text in the results and discussion sections to incorporate these new results, above reference, and the question on how to encourage innovation. We believe that they add further specificity to our manuscript and add to the overall theme of our publication that insights into innovation cannot be easily generalized.

3. I'd also like to see a more nuanced discussion of how pleiotropy fits into this study--a gene may already be established as "well-studied" for its association with a particular disease, but discovery of a novel connection between that gene and another disease can be just as impactful as an association between a novel gene and that disease (if not more so because we likely already know much about that well-studied gene's biology so it is potentially better poised for translational applications).

The reviewer’s insight on the identification of novel gene/disease associations is very important as it represents an extension of how innovation may be achieved for well-studied gene targets. We hence revised the discussion section to incorporate this broader understanding of innovation in human gene research.

Additionally, in our supplemental figure S4, where we previously only considered the presence of any phenotype, we now also include a corresponding analysis that scales the expectation in the literature according to the number of phenotypes in systematic mouse screens or in human genome-wide association studies. Interestingly, we find that our conclusions remain unaffected.

Minor comments:
Line 29: Consider also including Pfeiffer & Hoffman (2007) in this list [[https://urldefense.com/v3/__https://www.ncbi.nlm.nih.gov/pmc/articles/PMC1924584/__;!!Dq0X2DkFhyF93HkjWTBQKhk!Gf4eJAg3_wL_lSYqGL7CRwLdCtyHzN1tls-M6yZHQFcyF03Ja9U9mKu3yviykZ_6dx0o1-hGRUE$](https://urldefense.com/v3/__https:/www.ncbi.nlm.nih.gov/pmc/articles/PMC1924584/__;!!Dq0X2DkFhyF93HkjWTBQKhk!Gf4eJAg3_wL_lSYqGL7CRwLdCtyHzN1tls-M6yZHQFcyF03Ja9U9mKu3yviykZ_6dx0o1-hGRUE$) ].

We now include the reference on the former line 29 (now line: 30), and on every further appearance of the corresponding group of references.

Lines 84-86: apologies for being pedantic, but this isn't strictly true--the "full space of the possibly explorable entities" has only been truly available after the recently published telomere-to-telomere sequencing paper, which found an additional ~2,200 paralogous gene copies and 115 protein-coding genes ([https://urldefense.com/v3/__https://www.biorxiv.org/content/10.1101/2021.05.26.445798v1__;!!Dq0X2DkFhyF93HkjWTBQKhk!Gf4eJAg3_wL_lSYqGL7CRwLdCtyHzN1tls-M6yZHQFcyF03Ja9U9mKu3yviykZ_6dx0o7lTL-F8$](https://urldefense.com/v3/__https:/www.biorxiv.org/content/10.1101/2021.05.26.445798v1__;!!Dq0X2DkFhyF93HkjWTBQKhk!Gf4eJAg3_wL_lSYqGL7CRwLdCtyHzN1tls-M6yZHQFcyF03Ja9U9mKu3yviykZ_6dx0o7lTL-F8$) ).

Precision is good and we thank the reviewer for pointing this out. We corrected the sentence, added the above reference, and further cite the initial publications on the human genome by Celera Genomics and The Human Genome Project.

Along the same lines, we additionally now specify in this sentence that it refers to human protein-coding genes (as the annotation of other genes continued and continues well beyond the initial publication of the drafts of the human genome).

Line 166: delete "is"

Done. Thank you.

Lines 294-298: The finding that HHMI-supported publications are enriched for early-stage research lends itself nicely to the argument that funding agencies can pursue high-risk, potentially high-reward research by "funding people not projects" ([https://urldefense.com/v3/__https://www.nature.com/articles/477529a__;!!Dq0X2DkFhyF93HkjWTBQKhk!Gf4eJAg3_wL_lSYqGL7CRwLdCtyHzN1tls-M6yZHQFcyF03Ja9U9mKu3yviykZ_6dx0o9JdX2ZI$](https://urldefense.com/v3/__https:/www.nature.com/articles/477529a__;!!Dq0X2DkFhyF93HkjWTBQKhk!Gf4eJAg3_wL_lSYqGL7CRwLdCtyHzN1tls-M6yZHQFcyF03Ja9U9mKu3yviykZ_6dx0o9JdX2ZI$) ). This argument would interface nicely with the discussion in lines 482-485, where the "people not projects" ethos might be particularly productive and valuable if targeted towards early-career researchers.

We added a paragraph to our discussion section to discuss “funding people not projects” where we now added above and further references. Specifically, our findings on funding mechanisms only allow for partial support of this hypothesis as our findings suggest that it only applies to (at least a subset of person-centered) research grants whereas project-based research that is funded through research contracts instead of grants seems to similarly enrich for early-stage research. This might also extend beyond innovation in early-stage research as, at least for the United States of America, the development of COVID-19 vaccines seems to have supported through contracts instead of research grants (e.g.: https://crsreports.congress.gov/product/pdf/IN/IN11560)

We thank the reviewer for pointing out the relevance to early-career researchers and now added within above paragraph one sentence that explicitly states so and references a recent PNAS paper that shows that early funding success can lead to even more funding success within the subsequent years. Thus, interventions targeted to early-career scientists might act catalytically.

Lines 309-310: "strong support" and "avoidance" imposes an unnecessary judgement--better to just say the focus on early-stage research among domain-specific funding agencies can differ across countries and can change over time as those agencies reassess and reprioritize the areas of research they will pursue in any given year. (Also line 493--"pursuit or avoidance of innovation" implies NCI-funded research is not innovative, which might be read as an affront to a massive sector of this manuscript's potential audience).

We rephrased both occurrences. For the first occurrence we use the suggested sentence.

The second occurrence now reads: “Opposing temporal and country-specific trends for a single domain of biology (such as cancer research) might even provide opportunity to identify processes underlying shifting policies. For instance, one may hypothesize that for some domains of biomedical research a focus on well characterized genes could become desirable if meaningful societal impact stems from translational research, early diagnosis, or disease prevention.”

Reviewer #3:

I am happy with the current shape of the manuscript.  I especially like the clear formulation and testing of hypotheses.

We thank the reviewer for the kind words.
